# Plasticity-related microRNA and their potential contribution to the maintenance of long-term potentiation

**DOI:** 10.3389/fnmol.2015.00004

**Published:** 2015-02-23

**Authors:** Brigid Ryan, Greig Joilin, Joanna M. Williams

**Affiliations:** ^1^Brain Health Research Centre, University of Otago, DunedinNew Zealand; ^2^Department of Anatomy, Otago School of Medical Sciences, University of Otago, DunedinNew Zealand

**Keywords:** long-term potentiation, microRNA, maintenance, synaptic plasticity, memory

## Abstract

Long-term potentiation (LTP) is a form of synaptic plasticity that is an excellent model for the molecular mechanisms that underlie memory. LTP, like memory, is persistent, and both are widely believed to be maintained by a coordinated genomic response. Recently, a novel class of non-coding RNA, microRNA, has been implicated in the regulation of LTP. MicroRNA negatively regulate protein synthesis by binding to specific messenger RNA response elements. The aim of this review is to summarize experimental evidence for the proposal that microRNA play a major role in the regulation of LTP. We discuss a growing body of research which indicates that specific microRNA regulate synaptic proteins relevant to LTP maintenance, as well as studies that have reported differential expression of microRNA in response to LTP induction. We conclude that microRNA are ideally suited to contribute to the regulation of LTP-related gene expression; microRNA are pleiotropic, synaptically located, tightly regulated, and function in response to synaptic activity. The potential impact of microRNA on LTP maintenance as regulators of gene expression is enormous.

## INTRODUCTION

Long-term potentiation (LTP) is a form of synaptic plasticity whereby high frequency stimulation (HFS) induces a long-lasting enhancement of synaptic transmission. LTP is widely accepted as an excellent model for the molecular mechanisms that mediate long-term information storage in the brain. Many of its key properties are analogous to those of long-term memory, including input specificity, rapid induction, and co-operativity (Abraham and Williams, 2008). Most importantly, LTP is persistent: indeed an *in vivo* study suggests that LTP can last for at least a year after induction (Abraham et al., 2002). Although the potential for persistent LTP was noted in the earliest studies (Bliss and Gardner-Medwin, 1973), we still do not understand how the mechanisms underlying the stabilization of synaptic change allow LTP to persist for periods of days or weeks.

MicroRNA (miRNA) are endogenous non-coding RNA that act as post-transcriptional inhibitors of protein synthesis. They function by base-pairing with miRNA response elements (MREs) located in target messenger RNA (mRNA). This occurs within the ribonuclear protein complex known as the RNA-induced silencing complex (RISC; Kawamata and Tomari, 2010). To date, 2588 unique mature human miRNA have been annotated (miRBase 21). MiRNA are predicted to regulate the activity of more than 60% of human protein-coding genes (Friedman et al., 2009), although others predict far fewer in humans (30%; Lewis et al., 2005) and in *Caenorhabditis elegans* (10%; Lall et al., 2006). Even considering the most conservative estimates, the potential impact of miRNA activity on protein expression is profound. MiRNA are present in all body tissues (Lee et al., 2008), and their stability in circulating fluids suggests that they play an important role in cell–cell communication (Chen et al., 2012) and have utility as biomarkers of disease (Etheridge et al., 2011). Further, specific miRNA are crucial for development and function of both neurons and glia (Sayed and Abdellatif, 2011) and miRNA dysfunction is associated with neurodegenerative diseases, including Alzheimer’s disease (AD), which is characterized by memory impairment (Delay et al., 2012; Kim et al., 2014).

While recent reviews have detailed miRNA regulation at synapses (Siegel et al., 2011; Sim et al., 2014), their potential role as biomarkers of neurological disease (Sheinerman and Umansky, 2013) and highlighted the involvement of miRNA in a wide range of synaptic processes, from neurotransmission (Higa et al., 2014) to morphology (McNeill and Van Vactor, 2012), miRNA function in LTP has not been addressed specifically. To address this gap in the literature, this review will summarize experimental evidence for the proposal that miRNA play a major role in the regulation of LTP.

### LONG-TERM POTENTIATION

LTP is not a unitary phenomenon: multiple forms exist distinguished by stimulation paradigm, experimental preparation and brain region. While LTP can be induced in the visual cortex and a variety of cortical and subcortical structures (Abraham et al., 2002; Dityatev and Bolshakov, 2005; Zhao et al., 2005; Cooke and Bear, 2014), the majority of LTP research focuses on the hippocampus, a memory-related structure with a robust circuitry that lends itself well to experimentation. Here, LTP can be triggered not only by electrical stimulation (e.g., theta-burst, delta-burst), activation of metabotropic glutamate receptors (mGluRs) and brain-derived neurotrophic factor/tropomyosin receptor kinase B (BDNF/TrkB) signaling (Bortolotto et al., 1994; Balschun et al., 1999; Huang et al., 2013; Schildt et al., 2013) but by learning itself (Whitlock et al., 2006). This, alongside the remarkable persistence of LTP at hippocampal perforant path-dentate gyrus synapses, provides compelling evidence for the involvement of LTP-type plasticity in memory function.

Long-term potentiation induced at perforant path synapses in awake freely moving animals consists of three temporally and mechanistically distinct canonical phases: LTP1, LTP2, and LTP3 (Abraham and Otani, 1991). LTP1 refers to the initial, rapid strengthening of synapses, independent of protein synthesis and lasting for a few hours at most (Racine et al., 1983; Abraham and Otani, 1991). The putative cellular mechanisms that underlie LTP1, including enhanced release of neurotransmitter (Bayazitov et al., 2007), protein kinase activation (Sacktor et al., 1993; Lisman et al., 2012), and trafficking of both α-amino-3-hydroxy-5-methyl-4-isoxazolepropionic acid (AMPA) and *N*-methyl-D-aspartate (NMDA) subtypes of glutamate receptors to synapses (Williams et al., 1998; Hayashi et al., 2000; Shi et al., 2001), are likely to only explain short term synaptic strengthening. In contrast, the latter two phases require new protein synthesis. LTP2, the intermediate phase, requires synaptically localized protein synthesis but not new gene transcription, and has an average decay time constant of a few days (Otani et al., 1989; Kang and Schuman, 1996). However, as dendrites cannot maintain LTP for long periods of time when physically separated from their cell bodies (Kang and Schuman, 1996), translation of extant mRNA alone cannot explain LTP stabilization. The most long-lasting phase, LTP3, is dependent on new gene transcription as well as translation and can last for periods of weeks when induced at perforant path synapses (Nguyen and Kandel, 1996; Abraham et al., 2002). Here, the gene expression changes that accompany LTP maintenance have been intensively studied (Abraham and Williams, 2003).

Indeed, recent microarray studies have identified many LTP-induced, co-regulated genes, supporting the concept that LTP persistence involves regulation of coordinated gene networks (Lee et al., 2005; Park et al., 2006; Wibrand et al., 2006; Havik et al., 2007). Our recent studies have shown that the LTP-related transcriptional response is not limited to periods of minutes following LTP but extends to hours and days (Ryan et al., 2011, 2012). Bioinformatic analysis of this data set using Ingenuity Pathway Analysis (IPA) algorithms predicts this ongoing transcriptional response contributes both to dynamic alteration of synapses, through regulation of calcium dynamics, protein kinases, and synaptogenesis, and higher level regulation of gene expression. Intriguingly, this analysis predicted that miRNA functioned as regulatory hubs in these networks. This result suggests that complex processes such as LTP, involving the coordinated regulation of gene networks, may require fine-tuning of protein synthesis by miRNA, acting alongside other translational regulators (Bramham and Wells, 2007; Sossin and Lacaille, 2010; Jung et al., 2014).

### MicroRNA BIOGENESIS AND FUNCTION: KEY CHARACTERISTICS RELEVANT TO LTP

We propose that miRNA are ideally suited to control the rapid, coordinated and region-specific translation that underlies LTP maintenance. The characteristics of miRNA biogenesis and function that are of particular interest in this regard are tight spatial and temporal control of miRNA expression, their ability to function in a combinatorial manner with other miRNA, and the ability of a single miRNA to coordinate the expression of many mRNA related by their MRE sequences.

#### microRNA function

Mature miRNA range from 15 to 34 nucleotides in length and their average length is 22 nucleotides (miRBase 21). The functional impact of miRNA binding depends primarily on the thermo-stability of base-pairing between the target mRNA MRE and the miRNA 5^′^-seed sequence (nucleotides 2–8; Bartel, 2009); the 3^′^ region of the miRNA affects function to a lesser degree (Doench and Sharp, 2004). MiRNA usually interact with MREs in the 3^′^-untranslated region (UTR) of target mRNA (Bartel, 2009); yet the 5^′^-UTR and protein-coding regions can also contain functional MREs (Lytle et al., 2007; Forman et al., 2008), though the effect may be marginal (Hafner et al., 2010). In animals, complementarity between miRNA and their mRNA targets is typically imperfect (Bartel, 2004); this creates an internal bulge structure that distorts the helix, thereby preventing mRNA cleavage but allowing translational repression (Zeng et al., 2003; Rana, 2007). The exact mechanisms of RISC-induced translational repression are still under debate (reviewed by Morozova et al., 2012), however, translational repression is generally coupled ultimately with mRNA degradation via deadenylation (Eulalio et al., 2009) and decapping (Behm-Ansmant et al., 2006). Infrequently, imperfect miRNA:mRNA base-pairing can cause translational repression without mRNA degradation, which may be reversible (Bhattacharyya et al., 2006; Schratt et al., 2006). In contrast, perfect or near-perfect base-pairing can result in mRNA degradation via endonucleolytic cleavage, but this is very rare in animals (Yekta et al., 2004). Intriguingly, miRNA have been shown to up-regulate translation under some circumstances in mammals (Vasudevan et al., 2007; Orom et al., 2008).

#### microRNA biogenesis

**FIGURE 1:**
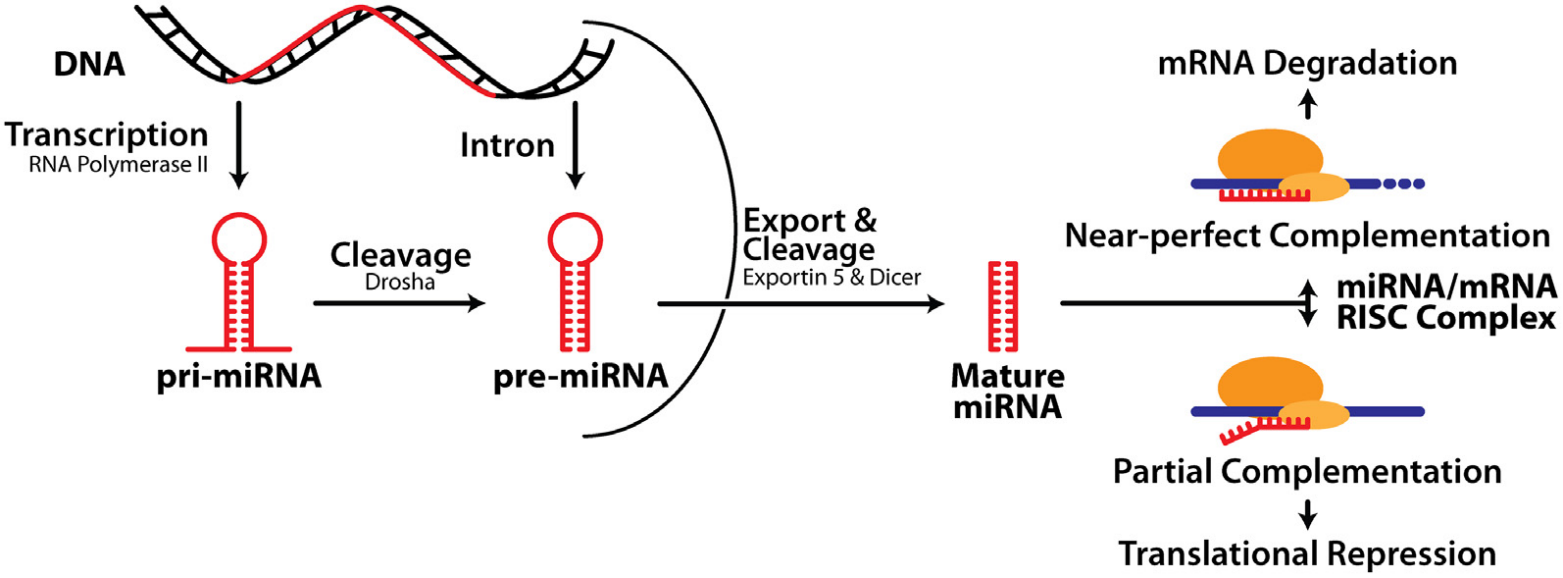
**MicroRNA biogenesis and mechanism of action.** See text for details.

MiRNA biogenesis consists of three major processes: transcription of a much longer primary transcript (pri-miRNA), followed by two consecutive cleavage events initiated by the RNase III enzymes Drosha and Dicer, which generate a precursor miRNA (pre-miRNA) and ultimately liberate the mature miRNA (**Figure 1**). Note that both the 5^′^ or 3^′^ strands of the miRNA hairpin can release a functional miRNA and these are denoted as, e.g., miR-132-5p or miR-132-3p respectively. Precise control of miRNA expression can be modulated at multiple points throughout this pathway (Thomson et al., 2006). In addition, pri- and pre-miRNA can be modified via RNA editing, affecting their activity (Peng et al., 2012). There is also evidence that turnover of some neuronal miRNA is activity-dependent (Krol et al., 2010a), and tightly regulated (reviewed in Krol et al., 2010b), although this is less well understood than miRNA biogenesis.

#### Tight spatial and temporal control

As miRNA are functional immediately after transcription and processing, they can respond more rapidly to external stimuli than transcription factors, which require translation into protein, transportation to the nucleus, and often post-translational modification to regulate gene expression. Tight spatial and temporal control of miRNA activity could allow miRNA to regulate translation selectively at individual synapses. This hypothesis is supported by the observation that both pri- and pre-miRNA, as well as their cleavage partners Drosha, DGCR8 and Dicer, are located at mouse hippocampal post-synaptic densities (PSDs; Lugli et al., 2005, 2012). Under normal conditions, Dicer was shown to be inactive in the PSD; however, following synaptic activation, the release of calcium caused calpain, a proteolytic enzyme, to activate Dicer, thereby triggering the conversion of pre-miRNA into active, mature miRNA (Lugli et al., 2005). Furthermore, pri-miRNA were found to be highly enriched with RNA transport granules, suggesting they can be transported alongside mRNA and processed at the PSD (Lugli et al., 2012). These important studies suggest that the expression of mature miRNA can be locally regulated by activity, providing a possible mechanism for translational regulation specific to activated synapses, thus contributing to the input specificity of LTP.

#### microRNA are highly pleiotropic

Individual miRNA can coordinately regulate the translation of many mRNA that share the same or similar MRE sequences, as complete complementation is not required for miRNA activity (Krek et al., 2005). Indeed, Selbach et al. (2008) demonstrated that one miRNA can not only promote degradation of hundreds of mRNA transcripts, but also repress the production of hundreds of additional proteins at the level of translation. Furthermore, the short sequences involved in miRNA:mRNA interactions make them well-suited for combinatorial effects with other miRNA or RNA-binding proteins that associate with separate sites on the same target mRNA (Krek et al., 2005). There is experimental evidence that multiple miRNA regulate the expression of the activity related cytoskeletal protein (Arc); in some cases in an additive manner (Wibrand et al., 2012). Additionally, there is evidence that miRNA act co-operatively with fragile X mental retardation protein (FMRP), an RNA-binding protein, known to regulate the translation of plasticity-associated mRNA (Xu et al., 2008). FMRP inhibits translation of Arc and CaMKIIα (calcium/calmodulin-dependent protein kinase IIα; Bassell and Warren, 2008) and *Fmr1* knockout mice exhibit impaired LTP at CA1 synapses (Lauterborn et al., 2007). FMRP is also associated with a number of miRNA in the mouse brain, including miR-132-3p, miR-125b-5p, miR-138-5p, and miR-124-3p (Edbauer et al., 2010), the last mediated by the FMRP Drosophila homolog dFMR1 to inhibit dendritic arbor (Xu et al., 2008). Furthermore, mouse models of fragile X syndrome showed regulation of miR-9a-5p and miR-124-3p by the FMRP family protein, FXR1P, which forms a complex with Dicer and affects miRNA processing (Xu et al., 2011). Interestingly, FXR1P has been found to be subjected to miRNA inhibition, creating a feedback loop that is becoming more evident in gene networks (Cheever et al., 2010). These feedback loops imply a homeostatic role for miRNA. It is noteworthy that many miRNA targets are regulatory genes such as transcription factors (Tsang et al., 2007) thus extending the range of genes regulated by one miRNA beyond those that it interacts with directly.

## MicroRNA ARE REGULATED IN RESPONSE TO LONG-TERM POTENTIATION

If miRNA regulate LTP-related protein synthesis, we would expect to see a change in the level of active, mature miRNA in response to LTP induction. LTP induced *in vitro* has been shown to regulate miRNA levels (Park and Tang, 2009; Lee et al., 2012a), however, the Bramham laboratory was the first to investigate miRNA expression after LTP induction in the dentate gyrus *in vivo* (Wibrand et al., 2010, 2012). Using anesthetized rats, they confirmed differential expression of three miRNA in dentate gyrus tissue 2 h after induction: miR-132-3p and miR-212-3p were up-regulated, and miR-219a-5p was down-regulated. Surprisingly, when HFS was delivered in the presence of an NMDA receptor antagonist expression of all three regulated miRNA was enhanced, despite ablation of LTP, suggesting that NMDA receptor activity represses miRNA levels. In contrast, regulation of the miRNA was blocked by a group 1 mGluR antagonist, which also prevented activity-dependent depotentiation, leading to the conclusion that differential expression of miRNA was functionally correlated with reversal of LTP.

By contrast, our recent work using an *in vivo* awake rat model (Abraham et al., 2002; Bowden et al., 2012) and Affymetrix miRNA arrays showed that at 20 min post-LTP the majority of the 65 differentially expressed mature miRNA transcripts were down-regulated, including miR-132-3p and miR-34a-5p (Joilin et al., 2014). These data contrast with the work of Wibrand et al. (2010, 2012), who found no apparent rapid regulation of miR-34a-5p or miR-132-3p, and Pai et al. (2014), who reported rapid up-regulation of miR-34a-5p, with no change in miR-132-3p levels. These discrepancies may potentially be accounted for by differences in stimulation paradigms and normalization procedures. Our data, interpreted alongside the generalized up-regulation of mRNA transcripts 20 min post-LTP (Ryan et al., 2011, 2012), suggest that down-regulation of miRNA may contribute to the long-term changes that lead to LTP persistence by rapidly releasing inhibition of target mRNA transcripts. We have also quantified miRNA expression at later time points using our *in vivo* awake rat model. While miR-132-3p had returned to baseline by 5 h, miR-34a-5p remained down-regulated (Ryan et al., 2012; Joilin et al., 2014) alongside miR-24-3p (Ryan et al., 2012); a finding consistent with the prediction that miR-34a-5p and miR-24-3p target mRNA that are up-regulated 5 h post-LTP (Ryan et al., 2012). As expression of these miRNA returned to baseline by 24 h, these results support the hypothesis that translational suppression of mRNA is released during the late phase of LTP to allow consolidation of LTP. Interestingly, using reverse transcription-quantitative polymerase chain reaction (RT-qPCR), we showed that the observed down-regulation of miR-34a-5p and miR-132-3p was mediated by NMDA receptors. Indeed, NMDA receptor antagonism in combination with tetanic stimulation led to a highly variable increase in miR-34a-5p, revealing an accord with Wibrand et al. (2010) and suggesting that in awake animals, the NMDA receptor-mediated reduction of miRNA levels out-competes an independent process working to increase them.

Very recently, Pai et al. (2014) have questioned the assumption that the quantity of total miRNA accurately represents miRNA activity. Instead, they have quantified miRNA bound to Ago2, one of the four Argonaute family proteins that can anchor mature miRNA within the RISC and allow miRNA activity. Using Ago2 immunoprecipitation, locked nucleic acid (LNA)-based microarrays and RT-qPCR validation, they demonstrated differential expression of Ago2-bound miRNA after LTP induction in the dentate gyrus of anesthetized rats (see **Table 1** for details). This differential expression was quantitatively and qualitatively different to that measured in whole dentate gyrus lysate. Ago2 immunoprecipitate/whole lysate ratios indicated that eight miRNA associated with Ago2 in response to LTP induction, while three miRNA dissociated from Ago2 in response to LTP induction.

**Table 1:** Differential expression of miRNA after LTP induction.

Study	Organism	Preparation	LTP induction	Time-points post-LTP induction	Identification of candidate miRNA	Control	Criteria for differential expression	Differentially expressed miRNA determined by RT-qPCR validation
Joilin et al. (2014)	4–6 months old Sprague Dawley rat	Dentate gyrus; LTP *in vivo*, un-anesthetized	HFS to PP-GC synapses	20 min, 5 and 24 h	Affymetrix microarray: 65/6,703 miRNA had FC ≥ 1.15; *n* = 4	Unstimulated hemisphere (within animal)	FC ≥ 1.15; *p* < 0.05: paired *t*-test	miR-34a-5p down-regulated at 20 min (FC = 0.44 ± 0.07; *n* = 8) and 5 h (FC = 0.53 ± 0.16; *n* = 5); back to baseline at 24 h miR-132-3p down-regulated at 20 min (FC = 0.37 ± 0.11; *n* = 8); back to baseline at 5 h and 24 h
Lee et al. (2012a)	4–5 weeks old Sprague Dawley rat	Hippocampal slice	Chemical LTP	30, 60, 120 min	Agilent microarray: 5/287 miRNA had FC ≥ 1.5 at at least one time-point; *n* = 1	Slices that did not undergo LTP induction	FC ≥ 1.5; *p* < 0.05: one-way ANOVA and LSD *post hoc*	miR-188-5p up-regulated at 60 min (FC = 1.75 ± 0.3; *n* = 3); no regulation at 30 min or 120 min. Four other miRNA (unidentified) were not regulated at any time-points
Pai et al. (2014)	Adult Sprague Dawley rat	Dentate gyrus; LTP *in vivo*, anesthetized. Total lysate and Ago2 IP examined	HFS to PP-GC synapses	30, 120 min	miRCURY microarray: 44/376 miRNA differentially expressed (*p* < 0.05: Student’s *t*-test with Dunn–Bonferroni correction); *n* = 3	Unstimulated hemisphere (within animal)	*p* < 0.05; one-way ANOVA	Total lysate: miR-384-3p, miR-29b-3p, miR-219a-5p, miR-592-5p, miR-20a-5p, let-7f-5p, miR-338-3p, miR-212-3p, miR-34a-5p, miR-19a-3p, miR-326-3p up-regulated at 30 min; miR-223-3p down-regulated at 30 min. Ago2 IP: miR-384-3p, miR-29b-3p, miR-219a-5p, miR-592-5p, miR-20a-5p, let-7f-5p, miR-338-3p, miR-330-5p, miR-223-3p, miR-34a-5p, miR-19a-3p up-regulated at 30 min; miR-212-3p down-regulated at 30 min
Park and Tang (2009)	7–8 weeks old C57/BL6 mice	Hippocampal slice	Chemical LTP	0, 15, 30, 60, 120 min	GenoExplorer microarray: 55/237 miRNA had mean FC over all time-points ≥ 2; *n* = 1	Slices that did not undergo LTP induction	FC ≥ 2	miR-181b-5p not regulated at any time-point (*n* = 10); miR-128a-3p not regulated at any time-point (*n* = 10)
Ryan et al. (2012)	4–6 months old Sprague Dawley rat	Dentate gyrus; LTP *in vivo*, un-anesthetized	HFS to PP-GC synapses	5 h	IPA network analysis: miRNA identified as hubs in LTP-related gene networks	Unstimulated hemisphere (within animal)	FC ≥ 1.15; *p* < 0.05: paired *t*-test	miR-34a-5p down-regulated (FC = 0.53 ± 0.16; *n* = 5); miR-24-3p down-regulated (FC = 0.59 ± 0.12; *n* = 9)
Wibrand et al. (2010)	Adult Sprague Dawley rat	Dentate gyrus; LTP *in vivo*, anesthetized	HFS to PP-GC synapses	10, 120 min	LC Sciences microarray: 21/237 miRNA had FC ≥ 1.2 at 120 min; *n* = 2	Unstimulated hemisphere (within animal)	FC ≥ 1.20; *p* < 0.05: *t*-test	miR-132-3p up-regulated at 120 min (FC = 1.38; *n* = 8); miR-212-3p up-regulated at 120 min (FC = 1.26; *n* = 8); miR-219a-5p down-regulated at 120 min (FC = 0.68; *n* = 8). None of the three miRNA were regulated at 10 min (*n* = 5)
Wibrand et al. (2012)	Adult Sprague Dawley rat	Dentate gyrus; LTP *in vivo*, anesthetized	HFS to PP-GC synapses	30, 120 min	Arc-targeting miRNA	Unstimulated hemisphere (within animal)	FC ≥ 1.20; *p* < 0.05: *t*-test	miR-132-3p up-regulated at 120 min (FC = ∼1.6; *n* = 5); not regulated at 30 min. No regulation of miR-19a-3p, miR-34a-5p, miR-326-3p, miR-193a-3p at 30 min or 120 min

Ago2 IP, Argonaute 2 immunoprecipitates; FC, fold change; GC, granule cell; h, hour; HFS, high frequency stimulation; IPA, Ingenuity Pathway Analysis; LSD, least significant difference; LTP, long-term potentiation; min, minute; PP, perforant path; RT-qPCR, real time-quantitative polymerase chain reaction. Data are presented as mean ± SEM.

Collectively, these data (summarized in **Table 1**) demonstrate that individual miRNA are regulated between 20 min and 5 h after LTP induction, supporting the theory that miRNA mediate LTP-related protein synthesis. There is little overlap in the miRNA found to be differentially expressed in these seven studies, this may however be due to variation in the models used to induce LTP or differences in statistical analyses. MiR-34a-5p and miR-132-3p are the only miRNA that have been reported as differentially expressed in response to LTP by multiple laboratories. One study characterized the plasticity properties of a miR-132-3p/miR-212-3p double knockout mouse, and reported enhanced theta burst LTP in hippocampal slices, but no effect on tetanic LTP (Remenyi et al., 2013). Expression of both mRNA and protein products of potential miR-132-3p targets was unchanged, which may explain why tetanic LTP was not affected.

## MicroRNA REGULATE LTP-RELATED GENES

**FIGURE 2:**
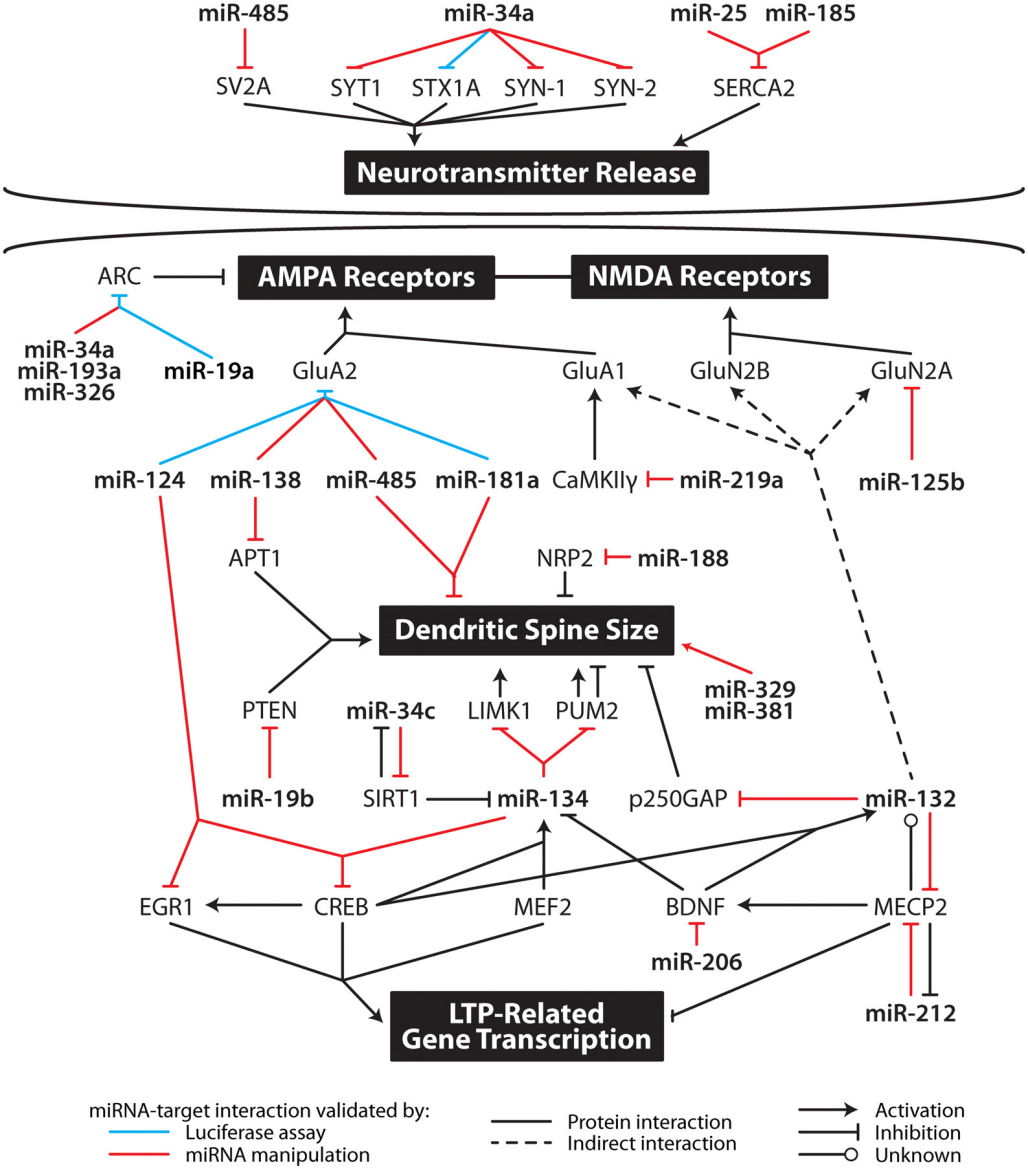
**Multi-level contribution of microRNA to synaptic plasticity.** MicroRNA likely influence the translation of multiple mRNA important in specific aspects of synaptic plasticity including neurotransmitter release, AMPA and NMDA receptor subunit levels, dendritic spine size, and gene transcription. APT1, acyl-protein thioesterase 1; ARC, activity-related cytoskeleton-associated protein; BDNF, brain-derived neurotrophic factor; CaMKIIγ, calcium/calmodulin-dependent protein kinase II gamma; CREB, cAMP response element-binding protein; EGR1, early growth response 1; GluA1, glutamate receptor, ionotropic, AMPA 1; GluA2, glutamate receptor, ionotropic, AMPA 2; GluN2A, glutamate receptor, ionotropic, NMDA 2A; GluN2B, glutamate receptor, ionotropic, NMDA 2B; LIMK1, LIM domain kinase 1; MECP2, methyl CpG binding protein 2; MEF2, myocyte enhancer factor-2; NRP2, neurophilin 2; PTEN, phosphatase and tensin homolog; PUM2, pumilio homolog 2; SERCA, sarco/endoplasmic reticulum Ca^2+^ ATPase; SIRT1, sirtuin 1; STX1A, syntaxin 1a; SV2A, synaptic vesicle glycoprotein 2A; SYN1, synapsin I; SYN2, synapsin II; SYT1, synaptotagmin I.

The putative molecular mechanisms underpinning LTP maintenance include enhanced release of neurotransmitter, alongside enhanced post-synaptic responsiveness driven initially by activation of protein kinases and glutamate receptor trafficking, which is ultimately underpinned by altered gene expression and structural reorganization of synaptic connections (Abraham and Williams, 2003; Lynch, 2004). The following sections discuss the mounting evidence suggesting that individual miRNA interact with transcripts coding for proteins important to all aspects of the maintenance of LTP. These studies are summarized in **Figure 2** and **Table 2**.

**Table 2:** Evidence for miRNA regulation of LTP-related genes.

Function	miRNA	mRNA	miRNA:mRNA interaction	Further information	Reference
Pre-synaptic vesicle release	miR-485-5p	SV2A	Direct inhibition *in vitro*	MiR-485-5p decreased the number of vesicles released after depolarization	Cohen et al. (2011)
	miR-34a-5p	SYN1, SYN2	Inhibition *in vitro*	MiR-34a-5p down-regulated 20 min (Joilin et al., 2014) and 5 h after LTP induction (Ryan et al., 2012); not regulated 30 min or 2 h after LTP induction (Wibrand et al., 2012)	Agostini et al. (2011)
	miR-34a-5p	SYT1, STX-1A	Direct inhibition *in vitro*	Increased miR-34a-5p correlated with decreased SYT1 and STX-1A in human Alzheimer’s disease (AD) brain. MiR-34a-5p down-regulated 20 min (Joilin et al., 2014) and 5 h after LTP induction (Ryan et al., 2012); not regulated 30 min or 2 h after LTP induction (Wibrand et al., 2012)	Agostini et al. (2011)
	miR-25-3p miR-185-5p	SERCA2	Decreased miR-25-3p and miR-185-5p correlated with increased SERCA2 *in vivo*	Decreased miR-25-3p and miR-185-5p linked to increased LTP in Schizophrenia mouse model	Earls et al. (2012)
Glutamate receptor regulation	miR-34a-5p miR-326-3p miR-19a-3p miR-193a-3p	Arc	Direct inhibition *in vitro*	MiR-34a-5p, miR-326-3p, miR-19a-3p, miR-193a-3p not regulated 30 min or 2 h after LTP induction. MiR-34a-5p down-regulated 20 min (Joilin et al., 2014) and 5 h after LTP induction *in vivo* (Ryan et al., 2012); not regulated 30 min or 2 h after LTP induction (Wibrand et al., 2012)	Wibrand et al. (2012)
	miR-181a-5p	GluA2	Direct inhibition *in vitro* (minimal)	MiR-181a-5p reduced spine volume and density in hippocampal neurons	Edbauer et al. (2010), Saba et al. (2012)
	miR-138-5p	GluA2	Inhibition *in vitro*	MiR-138-5p reduced spine volume in hippocampal neurons (APT-1 dependent)	Siegel et al. (2009)
	miR-485-5p	GluA2	Inhibition *in vitro*	MiR-485-5p reduced spine density and increased immature spines in hippocampal neurons	Cohen et al. (2011)
	miR-124-3p	GluA2	Direct inhibition *in vitro* (minimal)	–	Edbauer et al. (2010)
	miR-132-3p	GluA1, GluN2A, GluN2B	Indirect up-regulation *in vitro*	MiR-132-3p up-regulated 2 h after LTP induction (Wibrand et al., 2010, 2012); miR-132-3p down-regulated 20 min after LTP induction (Joilin et al., 2014)	Kawashima et al. (2010)
	miR-219a-5p	GluA1	Direct inhibition of CaMKIIy *in vivo*, which regulates GluA1	MiR-219a-5p down-regulated 2 h after LTP induction (Wibrand et al., 2010)	Kocerha et al. (2009)
	miR-125b-5p	GluN2A	Direct inhibition *in vitro*	MiR-125b-5p reduced spine width and increased length in hippocampal neurons (FMRP- dependent)	Edbauer et al. (2010)
	miR-212-3p	BDNF	Indirect inhibition via MeCP2 *in vivo*	–	Im et al. (2010)
	miR-132-3p	BDNF	miR-132-3p indirectly inhibits BDNF via MeCP2; BDNF indirectly up-regulates miR-132-3p via TrkB and ERK1/2	MiR-132-3p up-regulated 2 h after LTP induction (Wibrand et al., 2010, 2012); miR-132-3p down-regulated 20 min after LTP induction (Joilin et al., 2014)	Klein et al. (2007), Kawashima et al. (2010)
	miR-206-3p	BDNF	Direct inhibition *in vivo*	MiR-206-3p up-regulated in Tg2576 AD mice and human AD brain samples. Intraventricular injection of miR-206-3p antagomir increased BDNF and improved memory	Lee et al. (2012b)
Transcription	miR-132-3p	MeCP2	Reciprocal inhibition: MeCP2 inhibits miR-132-3p *in vitro*; miR-132-3p inhibits MeCP2 *in vivo*; MeCP2 KO decreased miR-132-3p *in vivo*	MiR-132-3p up-regulated 2 h after LTP induction (Wibrand et al., 2010, 2012); miR-132-3p down-regulated 20 min after LTP induction (Joilin et al., 2014)	Klein et al. (2007), Hansen et al. (2010), Im et al. (2010), Tognini et al. (2011)
	miR-212-3p	MeCP2	Reciprocal inhibition: MeCP2 inhibits miR-132 *in vitro*; miR-212-3p inhibits MeCP2 *in vivo*	MiR-212-3p up-regulated 2 h after LTP induction (Wibrand et al., 2010)	Im et al. (2010)
	miR-34c-5p	SIRT1	Direct inhibition *in vivo*	Increased miR-34c-5p correlated with decreased SIRT1 in memory impairment mouse models (APPS1-21 and aged)	Zovoilis et al. (2011)
	miR-124-3p	CREB, EGR1	Direct inhibition *in vitro*	–	Rajasethupathy et al. (2009), Yang et al. (2012)
	miR-134-5p	CREB	Direct inhibition *in vitro*	–	Gao et al. (2010)
Activity-dependent dendritogenesis	miR-134-5p	Pum2	Direct inhibition *in vitro*	Interaction only occurs after neuronal activity, not under basal conditions; miR-134-5p buffers Pum2 in a narrow range that is critical for activity-dependent dendritogenesis. MiR-134-5p decreased spine volume in hippocampal neurons	Schratt et al. (2006), Fiore et al. (2009)
	miR-134-5p	Limk1	Direct inhibition *in vitro*	Inhibition of Limk1 in dendrites relieved by BDNF	Schratt et al. (2006)
	miR-132-3p	p250GAP	Direct inhibition *in vitro* and *in vivo*	p250GAP regulates existing spine growth via Rac1 and kalirin-7; miR-132-3p affects spine size *in vivo*. MiR-132-3p up-regulated 2 h after LTP induction (Wibrand et al., 2010, 2012); miR-132-3p down-regulated 20 min after LTP induction (Joilin et al., 2014)	Impey et al. (2010), Mellios et al. (2011)
	miR-138-5p	APT1	Direct inhibition *in vitro*	MiR-138-5p and APT1 co-localized at the synapse; miR-138-5p reduced spine volume in hippocampal neurons (APT-1 dependent)	Banerjee et al. (2009), Siegel et al. (2009)
	miR-19b-3p	PTEN	Inhibition *in vitro*	MiR-19b-3p up-regulated after fear conditioning; miR-19b-3p increased total neurite length in hippocampal neurons	Kye et al. (2011)
	miR-188-5p	Nrp2	Direct inhibition *in vitro*	MiR-188-5p has no effect on dendritogenesis in hippocampal neurons MiR-188-5p is up-regulated 1 h after LTP induction (Lee et al., 2012a)	Lee et al. (2012a)

AD, Alzheimer’s disease; APT1, acyl-protein thioesterase 1; ARC, activity-related cytoskeleton-associated protein; BDNF, brain-derived neurotrophic factor; CaMKIIγ, calcium/calmodulin-dependent protein kinase II gamma; CREB, cAMP response element-binding protein; EGR1, early growth response 1; GluA1, glutamate receptor, ionotropic, AMPA 1; GluA2, glutamate receptor, ionotropic, AMPA 2; GluN2A, glutamate receptor, ionotropic, NMDA 2A; GluN2B, glutamate receptor, ionotropic, NMDA 2B; LIMK1, LIM domain kinase 1; LTP, long-term potentiation; MECP2, methyl CpG binding protein 2; NRP2, neurophilin 2; PTEN, phosphatase and tensin homolog; PUM2, pumilio homolog 2; SERCA2, sarco/endoplasmic reticulum Ca^2+^ ATPase; SIRT1, sirtuin 1; STX1A, syntaxin 1a; SV2A, synaptic vesicle glycoprotein 2A; SYN1, synapsin I; SYN2, synapsin II; SYT1, synaptotagmin I.

### MicroRNA REGULATE PRESYNAPTIC VESICLE RELEASE

MiRNA have been found within biochemical preparations that are enriched for presynaptic terminals (Xu et al., 2013). Intriguingly, three miRNA (miR-29a-3p, miR-99a-5p, miR-125a-5p) were shown to be released from synaptosomes in a calcium-dependent manner, and miR-125a-5p was shown to be endocytosed. Collectively, these findings led the authors to propose that miRNA release and uptake from nerve terminals may contribute to intercell communication. Other miRNA have also been implicated in the regulation of neurotransmitter release. MiR-25-3p and miR-185-5p target sarco/endoplasmic reticulum Ca^2+^ ATPase (SERCA2), which is involved in the maintenance of Ca^2+^ in the endoplasmic reticulum (Earls et al., 2012). Interestingly, SERCA2 is over-expressed in a mouse model of schizophrenia, concomitant with decreased miR-25-3p and miR-185-5p expression. As a result, calcium in the presynaptic terminal was increased, leading to increased neurotransmitter release and increased LTP. Restoration of these two miRNA attenuated the enhanced LTP.

Furthermore, two miRNA, miR-485-5p and miR-34a-5p, have been shown to interact with a number of transcripts that code for synaptic vesicle proteins (**Table 2**). Of these, synapsin 1a (SYN1), synaptotagmin I (SYT1), and syntaxin 1a (STX1A) have been implicated in LTP; SYN1 and SYT1 are regulated in response to LTP (reviewed in Abraham and Williams, 2003) and knockout of STX1A, a t-SNARE protein, impairs LTP in hippocampal slices (Fujiwara et al., 2006). While differential expression of miR-485-5p after LTP induction has not been reported, as outlined above, miR-34a-5p is down-regulated rapidly following LTP, the functional result of which may be to release inhibition of the synthesis of presynaptic vesicle proteins thereby contributing to enhanced release of neurotransmitter. Consistent with this hypothesis, miR-34a-5p is up-regulated in human AD, concomitant with SYT1 and STX1A down-regulation (Agostini et al., 2011).

### MicroRNA REGULATE GLUTAMATE RECEPTOR SUBUNITS

AMPA and NMDA glutamate receptors both play critical roles in LTP. Early work from our laboratory has shown that the levels of both AMPA and NMDA receptor subunits are dynamically regulated in the dentate gyrus in response to perforant path LTP (Williams et al., 1998, 2003, 2007; Kennard et al., 2009, 2014). Interestingly, the rapid up-regulation of AMPA receptors is not due to an increase in their synthesis, but to movement of receptor subunits into the membrane from an extrasynaptic pool (Shi et al., 1999; Williams et al., 2007; Granger et al., 2013). However, protein synthesis still plays an important role as it can affect NMDA receptor expression (Williams et al., 2007) putatively through the regulation of associated chaperone molecules. To date glutamate receptor subunits have been shown to be regulated by seven miRNA (miR-132-3p, miR-124-3p, miR-125b-5p, miR-138-5p, miR-181a-5p, miR-219a-5p, and miR-485-5p); including indirect up-regulation mediated through miR-132-3p (Kocerha et al., 2009; Siegel et al., 2009; Edbauer et al., 2010; Kawashima et al., 2010; Cohen et al., 2011; Saba et al., 2012). Recently, Wibrand et al. (2012) showed that a group of miRNA, including miR-34a-5p, regulates Arc, which is involved with trafficking AMPA receptors out of the synapse (Wibrand et al., 2012). These miRNA were not found to be regulated 30 min or 2 h after LTP induction; however, *in situ* hybridization and analysis of synaptoneurosome preparations showed that these Arc-targeting miRNA were synaptically expressed, suggesting basal inhibition of Arc.

MiRNA may also influence glutamate receptor subunit expression via regulation of BDNF, which is thought to contribute to the sustained structural and functional changes underlying LTP (Panja and Bramham, 2014). MiR-206-3p directly inhibits BDNF, and is up-regulated in AD (Lee et al., 2012b). Intra-ventricular injection of miR-206-3p antagomir increased BDNF expression in a mouse model of AD and improved memory function (Lee et al., 2012b). Additionally, miR-212-3p decreases BDNF protein expression in the dorsal striatum *in vivo* via inhibition of methyl CpG binding protein 2 (MeCP2; Im et al., 2010). *In vitro*, MeCP2 induces BDNF III mRNA expression and miR-132-3p decreases BDNF III mRNA expression in an MeCP2-dependent manner (Klein et al., 2007). This inhibition is significant as miR-132-3p inhibition can partially reduce BDNF-induced up-regulation of glutamate receptor subunits (Kawashima et al., 2010). Interestingly, BDNF has been shown to up-regulate miR-132-3p via the kinases TrkB and ERK1/2, suggesting a form of self-regulation through miRNA (Kawashima et al., 2010). These interactions between MeCP2, BDNF, and miR-132-3p/miR-212-3p are complex and need to be investigated further in the context of LTP. Together these data suggest that miRNA act co-operatively to regulate the expression of glutamate receptor subunits post-LTP.

### MicroRNA REGULATE LTP-RELATED TRANSCRIPTION FACTORS

While synthesis of proteins from extant mRNA is sufficient for LTP2, the concurrent and additional activation and regulation of transcription leads to the long-term stability of memory and LTP3 *in vivo*. Five miRNA (miR-34c-5p, miR-124-3p, miR-132-3p, miR-134-5p, and miR-212-3p) target proteins that regulate transcription in response to synaptic activity.

The BDNF-related gene MeCP2 is a putative dendritic growth regulator that mediates transcriptional repression. Li et al. (2011) have shown that a lack of activity-induced phosphorylation of MeCP2 causes enhanced LTP. As described above, both miR-132-3p and miR-212-3p interact with MeCP2 (Klein et al., 2007; Im et al., 2010). Excluding the role of BDNF in the circuit, there is evidence of reciprocal inhibition between MeCP2 and miR-132-3p/212-3p (Klein et al., 2007; Hansen et al., 2010; Im et al., 2010; Tognini et al., 2011). Furthermore, MeCP2 knockdown in HEK293 cells increased miR-212-3p and miR-132-3p expression (Im et al., 2010). This conflicts with other evidence showing MeCP2 knockout *in vivo* decreased miR-132-3p levels (Klein et al., 2007), but may be explained by the different preparations used. Further studies are needed to investigate these complex interactions involving MeCP2 in the context of LTP.

Another transcriptional repressor linked to LTP is Sirtuin 1 (SIRT1; Michan et al., 2010) which can be directly inhibited by miR-34c-5p (Zovoilis et al., 2011). In a study comparing young and aged mice, endogenous miR-34c-5p decreased SIRT1 protein in the hippocampus of aged, but not young, mice *in vivo* (Zovoilis et al., 2011). Over-expression of miR-34c-5p in the young mice caused a decrease in SIRT1 protein. Furthermore, in two memory impairment mouse models (aged and APPS1-21), an increase in miR-34c-5p was correlated with decreased SIRT1 protein (Zovoilis et al., 2011). Intrahippocampal injection of miR-34 inhibitors restored the level of SIRT1 protein in the APPS1-21 mice. The finding that miR-34c-5p is differentially expressed in a mouse model of AD supports the hypothesis that miR-34c-5p down-regulation is necessary for memory. In keeping with this hypothesis, increased miR-34c-5p expression was correlated with impaired contextual fear conditioning in mouse models of aging and AD and was rescued with miR-34c seed inhibitors (Zovoilis et al., 2011). However, while SIRT1 was the only target of miR-34c-5p that was investigated in this study, the authors note that other targets of miR-34c-5p are likely to be involved.

Interestingly, while miR-34c-5p regulates SIRT1, SIRT1 itself regulates miR-134-5p. In mouse neural cells, knockdown of SIRT1 increased miR-134-5p expression, which in turn inhibited cAMP response element binding protein (CREB) protein expression (Gao et al., 2010). *In vivo* miR-134-5p over-expression in the mouse hippocampus mimicked this effect (Gao et al., 2010). Furthermore, miR-134-5p is also regulated by an activity-dependant transcription factor, myocyte enhancer factor-2 (Mef2), which negatively regulates the number of excitatory synapses in mature hippocampal neurons (Flavell et al., 2006). Endogenous Mef2 is required for the depolarization-induced transcription of the miR-379-410 cluster, which contains miR-134 (Fiore et al., 2009). Inhibition of Mef2 in rat hippocampal neurons followed by depolarization caused a decrease in the precursors of miR-134-5p, which may lead to decreased mature miR-134-5p, affecting its ability to regulate its targets such as CREB. However, these interactions of miRNA and memory-related transcription regulators are yet to be studied in an LTP paradigm.

As a key regulator of activity-dependent dendritic morphogenesis, CREB mediates LTP-induced transcription, in part though the activation of an array of other transcription factors, which bind to specific response elements in immediate early genes (IEGs) such as early growth response 1 (EGR1). Both CREB and EGR1 have been linked to miRNA. MiR-124-3p directly decreased EGR1 expression *in vivo* (Yang et al., 2012) and CREB expression *in vitro* (Rajasethupathy et al., 2009). MiR-134-3p directly decreased CREB expression *in vitro* (Gao et al., 2010). These miRNA:mRNA interactions are intriguing, but are yet to be validated in a mammalian LTP model.

### MicroRNA REGULATE DENDRITOGENESIS

The majority of excitatory synapses in the mammalian brain are formed on specialized protrusions from dendrites, known as spines. Dendritic spines exhibit actin-dependent morphological plasticity and their size has been correlated with synaptic strength (Matsuzaki et al., 2004). LTP3 consolidation, and long-term storage of memories, may be achieved by an increase in the size, and therefore strength, of potentiated synapses. Dendritic morphogenesis may allow synapses to maintain their enhanced strength for long periods, in spite of continual turnover of their constituent proteins. Specific miRNA regulate transcripts that code for proteins involved in the regulation of dendritic spine morphology, which may underlie LTP maintenance.

MiR-134-5p interacts with the translational regulatory protein, pumilio homolog 2 (Pum2), which mediates activity-dependent dendritogenesis. Pum2 is an RNA-binding protein that regulates translation and mRNA stability by binding the 3^′^UTR of mRNA targets. MiR-134-5p directly targets Pum2 in membrane-depolarized rat cortical neurons, but not under basal conditions, suggesting only newly processed miR-134-5p interacts with Pum2 (Fiore et al., 2009). Furthermore, miR-134-5p buffers Pum2 protein levels within a narrow range necessary for activity-dependent dendritogenesis (Fiore et al., 2009), suggesting that miR-134-5p-mediated regulation of Pum2 may be critical for the structural changes that underlie LTP maintenance. This proposal is supported by *in vivo* evidence (Christensen et al., 2010).

In contrast, miR-134-5p has been found to decrease spine volume in hippocampal neurons under normal conditions (Schratt et al., 2006). MiR-134-5p has been shown to interact with LIM domain kinase 1 (Limk1), a serine/threonine kinase that regulates actin polymerization by inactivating cofilin neurons (Schratt et al., 2006). Limk1 knockout mice exhibit dendritic spine structural abnormalities and enhanced LTP (Meng et al., 2002). Over-expression of miR-134-5p in normal mice impaired LTP persistence and performance in learning paradigms including fear conditioning, Morris water maze, and novel object recognition (Gao et al., 2010). Performance in these tasks and LTP persistence were rescued when miR-134-5p was knocked down. However, whether this rescue was mediated by Pum2 and/or Limk1, or other miR-134-5p targets is unknown.

MiR-132-3p regulates existing spine growth in neurons *in vitro* by directly inhibiting translation of p250GAP in a Rac1- and kalirin-7-dependent manner (Edbauer et al., 2010; Impey et al., 2010; Mellios et al., 2011). *In vitro* and *in vivo* studies suggest that the effect of miR-132-3p on spine morphology differs according to the developmental stage of the neuron: miR-132-3p triggers spine formation during the spine development phase, and increases the volume of existing spines once the majority of spines have developed (Edbauer et al., 2010; Hansen et al., 2010; Impey et al., 2010; Magill et al., 2010; Mellios et al., 2011; Tognini et al., 2011). Whether these effects of miR-132-3p on existing spines contribute past development to underlie LTP-related structural changes remains to be seen. Furthermore, over-expression of miR-132-3p leads to the formation of spines with low plasticity properties: the mushroom spines of stable mature spines, and immature filopodia (Mellios et al., 2011; Tognini et al., 2011; Tognini and Pizzorusso, 2012). This suggests that like miR-134-5p, an optimal range of miR-132-3p expression is required for plasticity-induced spine changes to occur.

MiR-138-5p has been found to decrease spine size in rat hippocampal neurons by directly targeting an enzyme co-localized at the synapse, called acyl-protein thioesterase 1 (APT1; Banerjee et al., 2009; Siegel et al., 2009). APT1 catalyzes the depalmitoylation of signaling proteins, a lipid modification that can affect not just the function of the protein but its localization; the latter is particularly important considering the extent of neuronal processes. By inhibiting APT1, miR-138-5p was found to increase the membrane localization of the G-protein Gα_13_, thus activating the downstream Rho signaling pathway, which has been implicated in spine morphology regulation (Tada and Sheng, 2006). MiRNA also regulate other pathways involved in spine morphogenesis, in addition to G-protein signaling pathways. MiR-19b-3p inhibits PTEN (phosphatase and tensin homolog), a modulator of the AKT-mTOR signaling pathway, which controls dendritic development and synapse formation (Kye et al., 2011). MiR-19b-3p was up-regulated in mouse CA1 tissue 3 h after fear conditioning and in cultured hippocampal neurons 1 h after NMDA stimulation (but not bicuculline stimulation). Based on this research, Kye et al. (2011) proposed that learning induces miR-19b-3p, which inhibits PTEN and prevents it dephosphorylating phosphoinositide-3-kinase (PI3K). This in turn releases inhibition of the mTOR pathway, leading to increased protein synthesis. In keeping with this theory, miR-19b-3p has been shown to increase total neurite length in hippocampal neurons (Kye et al., 2011). A number of other miRNA that are up-regulated in response to fear conditioning and neural stimulation are known to increase mTOR-dependent protein synthesis via PTEN or Fox01 regulation (miR-21-5p, miR-22-3p, miR-27a-3p, miR-106b-5p, miR-139-5p), but these miRNA were not investigated further in Kye et al.’s (2011).

As mentioned above, miR-188-5p is down-regulated 2 h after LTP induction in rat hippocampal slices (Lee et al., 2012a). In this study, it was found that miR-188-5p directly inhibited neuropilin 2 (Nrp2), a transmembrane receptor protein for class 3 semaphorins, which have been shown to be chemorepellents against axonal growth cones, and therefore may act in a similar way against growth cones for dendritic spines. An increase in Nrp2 induced a decrease in spine density in hippocampal neurons, but miR-188-5p application rescued this effect. Over-expression of miR-188-5p does not have the expected effect of increasing spine density; the authors suggested this may have been due to a saturation effect (Lee et al., 2012a).

Five other miRNA have been shown to regulate dendritic spine morphology in DIV 10–15 rat hippocampal neurons: miR-125b-5p (Edbauer et al., 2010), miR-181a-5p (Saba et al., 2012), miR-485-5p (Cohen et al., 2011), miR-329-3p, and miR-381-3p (Fiore et al., 2009). The latter study is particularly interesting, as it reported that miR-329-3p and miR-381-3p were necessary for activity-dependent increases in dendritic complexity, but application of these miRNA had no effect under basal conditions. This result suggests that these miRNA may play a role in activity-dependent dendritogenesis.

Collectively, these results indicate that a sub-set of miRNA regulates translation of several proteins that are involved in dendritic morphogenesis. While, in some cases, these interactions have been investigated in the context of LTP, research into the role of miRNA in dendritic morphogenesis has focused on the genesis of new spines in developing neurons. These same miRNA:mRNA interactions may also play a role in the growth of existing spines in adult neurons during LTP maintenance, but further research is required to test this hypothesis.

## DISCUSSION

This review has summarized current knowledge regarding the possible involvement of miRNA in LTP maintenance. We presented evidence that a subset of miRNA are differentially expressed between 20 min and 5 h after induction of LTP *in vivo* and converging lines of evidence suggesting that miRNA likely regulate the expression of proteins relevant to all aspects of LTP maintenance.

Key properties of miRNA – pleiotropism, tight regulation, synaptic localization, and responsiveness to activity – make them ideal candidates as regulators of the co-ordinated gene expression that underpins LTP maintenance. Some of these properties are shared with other non-coding RNA, which have also been implicated in the maintenance of memory (Mercer et al., 2008). The hypothesis that miRNA regulate LTP maintenance is underscored by the studies presented in this paper and clearly demonstrated in **Figure 2**, which shows the influence of individual miRNA on multiple LTP-related genes. For example, miR-132-3p has been linked to glutamate receptor expression, dendritogenesis and transcription factor regulation. In keeping with these results, miR-132-3p has been implicated in learning behavior *in vivo*: over-expression of miR-132-3p in mouse forebrain neurons was associated with deficits in novel object recognition (Hansen et al., 2010) and contextual fear conditioning increased pri-miR-132 expression (Nudelman et al., 2010).

There is strong evidence that miRNA fine-tune translation locally at activated synapses (Schratt et al., 2006; Bredy et al., 2011): miR-134-5p inhibits translation of Limk1 at the synapse until synaptic activation, at which point miR-134-5p is inactivated and Limk1 protein is expressed, leading to dendritic spine growth. Conversely, synaptic activation could trigger miRNA to suppress translation of synaptic proteins that prevent LTP maintenance. Either or both of these mechanisms would allow miRNA to couple synaptic activity to the selective protein synthesis that contributes to LTP2 and/or LTP3. This is consistent with the reported changes in miRNA expression post-LTP and the localisation of miRNA and proteins involved in miRNA biogenesis and function at synapses (Lugli et al., 2005, 2008; Schratt et al., 2006; Kye et al., 2007; Aschrafi et al., 2008; Siegel et al., 2009; Natera-Naranjo et al., 2010). There is, however, no direct evidence that miRNA affect local protein synthesis at the synapse and thereby contribute to the input specificity of LTP.

MiRNA may also affect the translational ability of mRNA transcripts as they are being transported from the soma to the axonal terminals or dendritic spines (Schratt et al., 2006). There is evidence that miRNA may be bound to mRNA in structures similar to processing-bodies (P-bodies) for transportation, allowing the miRNA to suppress translation of the mRNA until after synaptic activation (Schratt et al., 2006). The transcripts are likely to encode proteins that stabilize the changes at synapses that allow LTP to persist. For example, over-expression of miR-132-3p can result in increased levels of the NMDA receptor subunits GluN2A and GluN2B (Kawashima et al., 2010) and our own research has shown that post-LTP there is a rapid protein synthesis-dependent, transcription-independent increase in their expression (Williams et al., 2007).

That miRNA play a role in LTP-maintenance outside of the synapse is supported by evidence that miRNA influence the expression of LTP-related transcription regulators such as CREB, EGR1, MeCP2, SIRT1 and Mef2, contributing to the coordinate genomics response which underpins LTP3 by allowing tight regulation of a subset of genes post-LTP induction (Ryan et al., 2012; Joilin et al., 2014).

It is important to note that regulation of miRNA in response to LTP induction does not prove that they contribute to LTP maintenance. There are currently no reports of a direct effect of altering miRNA activity on LTP maintenance. Indeed, Dicer knockdown and concomitant down-regulation of miRNA had no effect on LTP (Konopka et al., 2010), however, this could be attributed to the short recording time of 50 min after HFS. By contrast, over-expression of individual miRNA *in vivo* prior to LTP induction was associated with a decrease in LTP (Gao et al., 2010; Scott et al., 2012), although these studies could not determine whether this affected LTP maintenance or LTP induction as the miRNA levels were altered prior to the induction of LTP. We also cannot rule out the possibility that miRNA regulation is occurring outside of neurons (e.g., in glia), or at synapses that are not undergoing LTP. Indeed, the observed changes in miRNA expression could be due to related forms of activity-dependent synaptic plasticity, such as LTD or depotentiation. For example, NMDA receptor-dependent LTD requires miR-191a-5p and miR-135a-3p for its maintenance (Hu et al., 2014), and electroconvulsive shock (ECS), which shares many properties with LTP, can induce differential expression of a large number of miRNA (Eacker et al., 2011). These forms of synaptic plasticity are likely to be activated concurrently to scale synapses in neighboring spines, dendrites, or neurons. Further research accounting for cell type and/or sub-cellular compartments is needed to resolve these issues.

An important next step for this field is to confirm that the miRNA:mRNA interactions presented here are indeed physiologically relevant to LTP maintenance, either *in vitro* or *in vivo*. Likewise, conditional regulation of miRNA levels or activity is required to discriminate between effects on the early and late phases of LTP. Furthermore, investigation of the mechanisms underpinning altered miRNA turnover and/or synthesis, particularly with regard to rapid down-regulation of miRNA is required. To establish the full cohort of LTP-related miRNA, high-throughput broad screens of differentially expressed miRNA at multiple time points after LTP induction are needed. Alongside this the development of improved target prediction algorithms would facilitate identification of relevant targets of these miRNA, and thereby increase our understanding of the functional significance of miRNA regulation in response to LTP induction.

## SUMMARY

We conclude that to date, there is insufficient evidence to confirm that miRNA contribute to the maintenance of LTP; seven studies from four groups have reported differential expression of miRNA in response to LTP induction and converging lines of evidence suggest that they are ideally suited to this purpose. The functional significance of miRNA in the maintenance of LTP remains to be determined; this is the next step in this challenging and exciting area of research.

## AUTHOR CONTRIBUTIONS

All authors were involved in the conception and writing of the review.

## Conflict of Interest Statement

The authors declare that the research was conducted in the absence of any commercial or financial relationships that could be construed as a potential conflict of interest.
